# Case report: A case of Paget disease outside the axillary breast

**DOI:** 10.1097/MD.0000000000037541

**Published:** 2024-03-29

**Authors:** Zhibing Zhou, Bing Zhou, Xiaobo Wu, Wensong Wei

**Affiliations:** The Third Department of Mammary Gland, Third Hospital of Nanchang, Nanchang, China.

**Keywords:** axilla, extramammary Paget disease

## Abstract

**Background:**

Extramammary Paget disease is a relatively rare and less malignant intraepithelial adenocarcinoma. t is found in areas with abundant distribution of apocrine sweat glands such as the external genitalia, external genitalia, and perianal area, with fewer armpits. The disease progresses slowly and is prone to misdiagnosis in clinical practice.

**Methods:**

We retrospectively analyzed a female patient. She had a left axillary mass for more than 2 years. Recently, the mass increased and the surface skin was ulcerated. Then she went to Jiangxi Provincial Dermatology Hospital for left axillary lesion resection, and the postoperative pathology showed Paget disease outside the breast. For further diagnosis and treatment, she came to our hospital. We diagnosed a tumor with uncertain or unknown dynamics in the left axillary breast. Under general anesthesia, left subaxillary mass resection, freezing and left breast cancer breast conserving surgery was performed.

**Results:**

The postoperative pathology of the left axillary mass combined with morphological and immunohistochemical results was consistent with Paget disease. Postoperative immunohistochemistry showed estrogen receptor (+, 20%), progesterone receptor (−), human epidermal growth factor receptor-2 (3+), Ki-67 (30%), cytokine7 (+), and p63 (−). Following up for 22 months, there has been no local recurrence, no swelling of the right axillary lymph node, no distant metastasis found on follow-up, and no complications such as upper limb lymphedema, upper limb sensory abnormalities, or motor disorders have been observed.

**Conclusion:**

Paget disease outside the axillary breast is relatively rare, and surgical resection is the best choice. The prognosis is good, and the recurrence rate is low.

## 1. Introduction

Extramammary Paget disease (EMPD) is a special type of cancerous disease. Because it often presents as eczema like, it is also known as eczema like cancer. This disease tends to occur in the elderly. The clinical manifestations are complex and atypical, which can easily lead to misdiagnosis and delayed treatment. Early pathological examination of skin lesions should be performed.

## 2. Case presentation

A 57 year old female patient presented with a left axillary mass for over 2 years. Two years ago, the patient had no obvious cause of a lump about the size of peanuts in the left armpit. The surface was red and swollen, without pain or tenderness, and there was no nipple discharge. Recently, the patient realized that the lump had increased and the surface skin was ulcerated. And then she went to Jiangxi Provincial Dermatology Hospital for left axillary lesion resection, and the postoperative pathology showed Paget disease outside the breast. For further diagnosis and treatment, she came to our hospital and was admitted by the outpatient department for a “left axillary mass.” The patient had a healthy past and no family members had a history of such illnesses.

**Figure 1.**
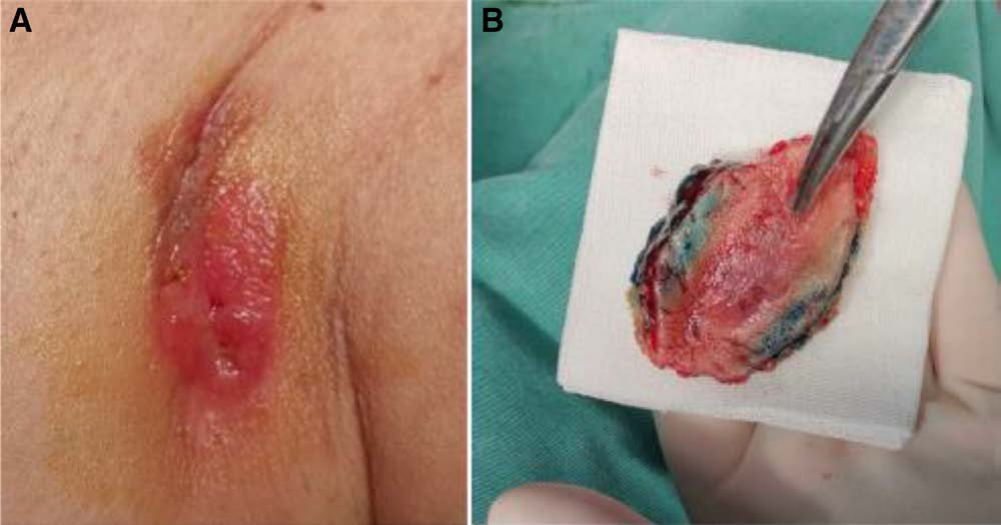
(A) A 3 cm × 2 cm area of redness and swelling can be seen in the left armpit, accompanied by ulceration and a small amount of secretion. (B) The intraoperative resection range, including diseased tissue, is approximately 7 cm × 5 cm.

Physical examination: The general condition was good, and there were no abnormalities in the system inspection. Specialized examination: Bilateral breasts are symmetrical, with average breast development. Bilateral nipples were normal and there was no nipple discharge. A red and swollen area of 3 cm × 2 cm in size could be seen in the left armpit, accompanied by ulceration and a small amount of secretions (Fig. 1A). A mass of approximately 3.0 cm × 2.0 cm in size could be palpated in the left armpit, which was hard and had a clear boundary, with moderate activity. No obvious abnormal mass could be palpated in the right breast, and no obvious enlarged lymph nodes could be palpated in the right armpit.

Laboratory and auxiliary examinations: Blood routine, coagulation, and bowel and bowel routine tests were all normal. No abnormalities were found in liver and kidney function, electrolytes, blood sugar, and blood lipids. The myocardial enzyme spectrum and tumor 7 items were all within the normal range. Hepatitis A, B, C, human immunodeficiency virus, and syphilis showed no significant abnormalities. Among the 5 items of thyroid function, anti thyroglobulin antibodies were 188.50 IU/mL, and anti thyroid microsomal antibodies were > 1000.0 IU/mL. The electrocardiogram indicated sinus rhythm and changes in some lead T waves. Chest and abdominal computed tomography plain scan showed several miliary lesions in both lungs and hepatic steatosis. No obvious abnormalities were found in the gallbladder, pancreas, spleen, kidneys, and bladder. No obvious abnormalities were found on plain and enhanced dual breast magnetic resonance imaging scans. Breast ultrasound indicates hypoechoic areas in both breasts, considering the possibility of breast adenosis, the United States of America breast imaging reporting and data system Class 2, bilateral lipoma, and left axillary lymph node enlargement. The postoperative pathology of Jiangxi Provincial Dermatology Hospital is consistent with Paget disease outside the armpit breast.

Upon admission, the diagnosis was a tumor with uncertain or unknown dynamics in the left axillary breast.

**Figure 2.**
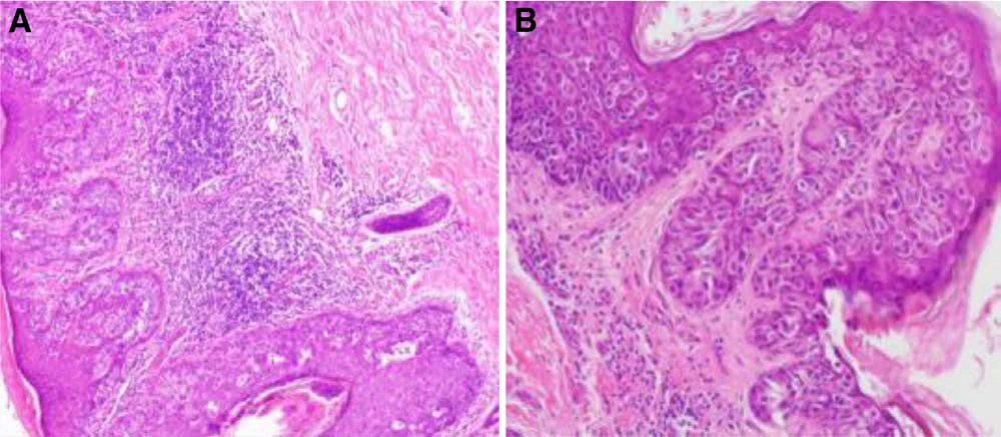
(A) A large number of atypical cells fill the squamous epithelium, infiltrate interstitial lymphocytes, and form germinal centers (hematoxylin-eosin stain; original magnification × 10). (B) Paget cells have clear boundaries, clear cytoplasm, delicate chromatin granules in the nucleus, and clear nucleoli (hematoxylin-eosin stain; original magnification × 20).

**Figure 3.**
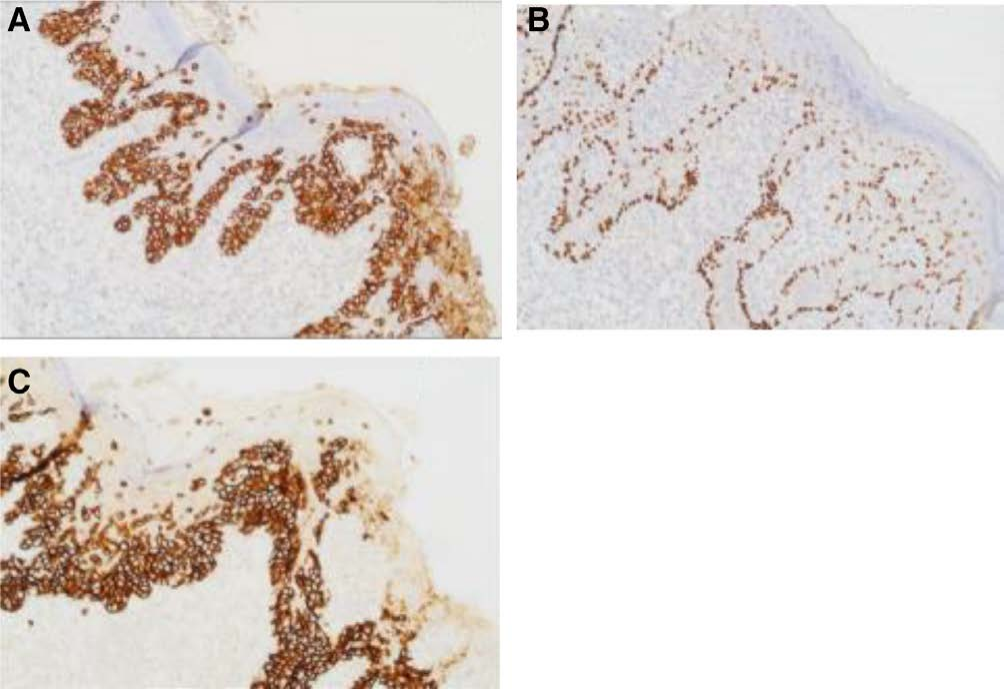
Immunohistochemical staining of the axillary Paget disease lesion (200 × magnification). (A) Positive staining for CK7; (B) Negative staining for p63; (C) Positive staining for C-erbB-2 (3+). C-erbB-2 = human epidermal growth factor receptor-2, CK = cytokine.

Treatment process: The patient was placed in a supine position and under general anesthesia, left subaxillary mass resection, freezing and left breast cancer breast conserving surgery was performed. We covered the affected area with gauze before cutting the skin and fix the skin. We made a shuttle shaped incision 1.5 cm outside the lesion area, approximately 7 cm in length, and separated toward the deep. During the surgery, the size of the tissue, including the lesion, was approximately 7 cm × 5 cm and frozen during the surgery (Fig. 1B). The results showed that fibrous adipose tissue can be seen in the left armpit, and a large number of inflammatory cells infiltrate. The diagnosis was to be confirmed by paraffin section. The postoperative pathology of the left axillary mass combined with morphological and immunohistochemical results was consistent with Paget disease. The pathological examination of the skin lesions showed an abundance of keratinization in the epidermis, a large number of atypical cells in the epithelial layer, infiltration of interstitial lymphocytes, and infiltration of Paget cells in the superficial dermis (Fig. 2). Postoperative immunohistochemistry showed estrogen receptor (+, 20%), progesterone receptor (−), human epidermal growth factor receptor-2 (3+), Ki-67 (30%), cytokine (CK)7 (+), and p63 (−) (Fig. 3). The patient incision healed well after surgery and did not undergo chemotherapy or radiotherapy. Regular follow-up and endocrine therapy were recommended for at least 5 years. Following up for 16 months, there has been no local recurrence, no swelling of the right axillary lymph node, no distant metastasis found on follow-up, and no complications such as upper limb lymphedema, upper limb sensory abnormalities, or motor disorders have been observed. She often shares her experiences with other patients and reminds them to seek early treatment if they have similar symptoms.

## 3. Discussion and conclusions

In 1874, James Paget first described a skin disease that occurred in women breasts, later known as Paget disease. In 1889, Crocker first reported Paget disease, also known as EMPD, which occurred on the male external genitalia. The name of this disease comes from its many similarities in clinical manifestations and histopathology with Paget disease of the breast. Extramammary Paget disease is a rare, low-grade malignant epithelial tumor within the epidermis, accounting for 6.5% of Paget disease. The usual onset age is between 50 and 80 years old.^[1]^

This disease can be divided into primary and secondary types based on the source of Paget cells. Primary EMPD is more common in areas with abundant sweat glands such as the external genitalia, external genitalia, and perianal area, and is less common in the armpit. It is most common in the female external genitalia (especially the labia majora), followed by the perianal area, male penis and scrotum. Only 1% to 2% of patients are located in the external auditory canal, umbilicus, eyelids, and armpits.^[1,2]^ Most skin lesions are localized and solitary, with a slow onset and a few cases involving multiple parts. Positive expression of mucin core protein suggests that the skin lesion is more likely to be multifocal EMPD, rather than the simultaneous occurrence of breast and extramammary Paget disease. Secondary EMPD is often skin metastasis of malignant tumors, such as rectal cancer, bladder cancer, endometrial cancer and gastric cancer. The pathogenesis of primary EMPD is not yet fully understood, and according to literature reports, it may be epidermal metastasis of sweat gland adenocarcinoma or a special type of skin in situ carcinoma. In the past, it was believed that Paget cell line sweat gland cancer spread along the ductal epithelium, but continuous sectioning revealed that lesions in the epidermal appendages were multifocal in origin, and in many cases, sweat gland duct and glandular lesions could not be found at all. Even if the epidermal lesions and sweat gland duct lesions were continuous, it was difficult to determine whether the expansion of the lesions was top-down or bottom-up.^[3]^ Histochemical studies have confirmed that Paget cells share the same apical secretory glandular like structure as sweat glands. Some scholars speculate that there may be similarities in histological origin between sweat gland adenocarcinoma and EMPD. EMPD is a special type of skin in situ cancer, where tumor cells originate from multi-directional differentiation potential cells in the skin and then spread to the mammary gland, hair follicles, sweat glands, and large sweat gland ducts below. Some scholars also believe that EMPD, sweat gland cancer, and other visceral tumors (such as rectal cancer, prostate cancer, kidney cancer, etc) originate from the same embryonic cells. EMPD skin lesions often manifest as multicentric erythema, exudation, erosion, desquamation, or eczema like lesions, which are easily confused with chronic skin diseases. However, long-term hormone and antifungal treatment has led to an expansion of the lesion area. Moreover, the axillary lymphatic vessels and blood vessels are abundant. If the cancer tissue invades the axillary blood vessels or nerves, it will increase the difficulty of surgery and even lose the opportunity for surgery. In the later stage of the tumor, there is obvious nodular damage, with increased itching. Secondary damage such as erosion, exudation, and scabbing can occur due to scratching. In rare cases, multiple skin lesions can occur simultaneously in the external genitalia and armpits.

Paget disease has the following typical histopathological features^[4]^: We can see excessive or incomplete keratinization of the epidermis; Single or nested Paget cells can be seen in the epidermis; Early Paget cells were located in the basal layer and above, and basal cells and surrounding keratinocytes were compressed and flattened; As the condition progresses, Paget cells can spread upwards, even to the entire layer of the epidermis, and Paget cells can also be seen in the stratum corneum; Paget cells are relatively large, with light stained cytoplasm and a circular or elliptical shape; Hair follicles and sweat gland ducts can also be affected; Paget cells can also exhibit invasive growth and enter the dermis; There is inflammatory cell infiltration in the superficial layer of the dermis, which can appear as a band. The commonly used positive markers for Paget cells include cerB-2, CK7, epithelial membrane antigen, and gross cystic disease fluid protein-15, which can help differentiate them from other diseases. Melanoma is different from Paget disease outside the breast, with positive expression of S-100 protein, HMB-45, melon-A, and MART-1. The tumor cells of Bowen disease do not express CKs such as gross cystic disease fluid protein-15, c-erb-2, carcinoembryonic antigen, epithelial membrane antigen, etc. CK7 and CK20 are valuable biomarker combinations that distinguish this disease from primary and secondary. In primary extramammary Paget disease, CK7 is often positive, while CK20 is negative, while CK7 and CK20 originating from the endoderm are both positive. It is worth noting that although CK7 positivity is helpful for the diagnosis of EMPD, CK7 positivity may also occur in Paget like Bowen disease and Paget like solar keratosis. However, in the pathological tissue of the former, poorly keratinized cells can be seen, while in the pathological tissue of the latter, solar elastic fiber degeneration can be seen in the dermis. This case has no history of breast disease or other tumors in the past. Based on pathology and postoperative immunohistochemistry of human epidermal growth factor receptor-2 (3+) and CK7 (+), it is considered as primary extramammary Paget disease. Chiu et al^[2]^ collected data on 7 patients with axillary EMPD over a period of 20 years (1989–2008), with an average age of 67.6 years (41–86) and a male to female ratio of 1:1.3. All patients had unilateral skin lesions, with 4 on the right side and 3 on the left side. The disease lasted for 2.3 years (1–3 years), with or without itching, presenting as chronic erythema to brown plaques. The lesions were extensively removed 2 to 3 cm outside, and there was no recurrence during follow-up (6–166 months). Including this case, there are 18 detailed reports in China involving axillary EMPD,^[5–7]^ including 13 males and 5 females. The average age is 68.6 years (47–89 years), with an average course of disease of 6.8 years. The lesions are mostly unilateral armpits, with only 3 cases having multiple lesions. Most early diagnoses are “eczema” or “tinea corporis,” and the final histopathological diagnosis considers EMPD. Immunopathology also showed that most CK7 were positive. Among them, 4 cases were complicated with other organ tumors. Compared with foreign countries, the incidence of male diseases in China is significantly higher than that in females, and the course of the disease is longer. This may be due to the rarity of this disease and atypical skin lesions, leading to long-term misdiagnosis. The other indicators are very similar, such as: the onset age is middle-aged and elderly people; All skin lesions are unilateral armpits; There are only a small number of concurrent local tumors. Wolf et al^[8]^ believe that EMPD can present various clinical manifestations, so it is often misdiagnosed initially. Therefore, when recurrent erosive erythema occurs in the armpit area and the treatment effect is not satisfactory, it is recommended to undergo biopsy and immunopathological chemical examination early to rule it out.

EMPD is mostly non invasive and limited to in situ carcinoma of the epidermis. There may also be adenocarcinoma of adjacent organs, such as rectal cancer, cervical cancer, or bladder cancer, which may metastasize to the epidermis of the corresponding part to cause secondary extramammary Paget disease, but rarely metastasize to local lymph nodes, especially distant organs.^[9]^ The recurrence rate of this type of EMPD after surgical resection is low, and the reasons for it are not only the clearer anatomical relationship between the axillary area and the genital area, but also other deeper reasons that need further research.^[2]^

In summary, EMPD occurring in the axillary region is relatively rare and often lacks typical symptoms, resulting in a high misdiagnosis rate. After diagnosis, surgical resection is the first choice, and for primary EMPD without metastasis, most cases can be completely cured. Although it has been observed from limited cases that it is mostly in situ cancer and rarely spreads or metastasizes, its multicenter origin and subclinical spread properties remain unchanged, so long-term follow-up is necessary after surgery.^[2]^ Once recurrence or metastasis is detected, immediate reoperation should be performed to improve patient survival rate.

## Author contributions

**Conceptualization:** Zhibing Zhou, Bing Zhou.

**Data curation:** Zhibing Zhou.

**Formal analysis:** Zhibing Zhou.

**Funding acquisition:** Zhibing Zhou.

**Investigation:** Zhibing Zhou.

**Resources:** Zhibing Zhou.

**Writing – original draft:** Zhibing Zhou.

**Writing – review & editing:** Zhibing Zhou, Bing Zhou, Xiaobo Wu, Wensong Wei.
